# Impact of Hypertension History and Blood Pressure at Presentation on Cardiac Remodeling and Mortality in Aortic Dissection

**DOI:** 10.3389/fcvm.2021.803283

**Published:** 2022-01-21

**Authors:** Matheus F. R. A. Oliveira, Walter E. M. Rocha, Julia D. Soares, Victor M. F. S. L'Armée, Mayara P. G. Martins, Aloísio M. Rocha, Audes D. M. Feitosa, Ricardo C. Lima, Pedro P. M. Oliveira, Lindemberg M. Silveira-Filho, Otavio R. Coelho-Filho, José R. Matos-Souza, Orlando Petrucci, Andrei C. Sposito, Wilson Nadruz

**Affiliations:** ^1^Department of Internal Medicine, School of Medical Sciences, State University of Campinas, São Paulo, Brazil; ^2^Pronto Socorro Cardiológico de Pernambuco, University of Pernambuco, Recife, Brazil; ^3^Department of Cardiology, Pontifical Catholic University of Campinas, Campinas, Brazil; ^4^Catholic University of Pernambuco Clinical Research Institute, Catholic University of Pernambuco, Recife, Brazil; ^5^Department of Surgery, School of Medical Sciences, State University of Campinas, São Paulo, Brazil

**Keywords:** aortic dissection, hypertension, mortality, left ventricular remodeling, blood pressure

## Abstract

**Objective:**

This study compared clinical, echocardiographic, and prognostic characteristics among patients with aortic dissection (AD) with (HypHist) and without (No-HypHist) hypertension history and evaluated the association of blood pressure (BP) at presentation with 1-year mortality, left ventricular (LV) remodeling and renal dysfunction.

**Methods:**

We investigated clinical and echocardiographic characteristics and 1-year mortality among 367 patients with AD (81% HypHist, 66% Type-A) from three Brazilian centers.

**Results:**

Patients with No-HypHist were more likely to have Marfan syndrome, bicuspid aortic valve, to undergo surgical therapy, were less likely to have LV hypertrophy and concentricity, and had similar mortality compared with HypHist patients. Adjusted restricted cubic spline analysis showed that systolic BP (SBP) and diastolic BP (DBP) at presentation had a J-curve association with mortality among patients with No-HypHist, but did not associate with death among patients with HypHist (*p* for interaction = 0.001 for SBP and = 0.022 for DBP). Conversely, the association between SBP at presentation and mortality was influenced by previous use of antihypertensive medications in the HypHist group (*p* for interaction = 0.002). Results of multivariable logistic regression analysis comprising the whole sample showed direct associations of SBP and DBP at presentation with LV hypertrophy (*p* = 0.009) and LV concentricity (*p* = 0.015), respectively, and an inverse association between pulse pressure at presentation and estimated glomerular filtration rate (eGFR) <60 ml/min/1.73 m^2^ (*p* = 0.008).

**Conclusion:**

Combined information on BP at presentation, previous diagnosis of hypertension, and use of antihypertensive medications might be useful to predict mortality risk and to estimate extra-aortic end-organ damage among patients with AD.

## Introduction

Patients with aortic dissection (AD) have high mortality risk and usually present high rates of alternative organ damage with prognostic value, including left ventricular (LV) remodeling and renal dysfunction (1–4). Hypertension is the leading and most common cause of AD (2) and is a major risk factor for myocardial remodeling and renal impairment in general populations (5, 6), raising the assumption that this condition could exert a relevant influence on the characteristics and prognosis among patients with AD.

Few studies have evaluated the impact of hypertension on clinical and prognostic features in AD, and available evidence suggests that hypertensive patients with AD are usually older and are less likely to receive surgical therapy, but have similar long-term mortality in comparison with those without hypertension (7, 8). Conversely, whether adverse cardiac remodeling differs between AD patients with and without hypertension is still uncertain. Importantly, the high rates of LV hypertrophy among patients with AD do not seem to be fully explained by hypertension itself (3, 9), suggesting that even AD patients without hypertension are at high risk of presenting adverse cardiac remodeling.

Blood pressure (BP) at presentation is a variable that is easily obtained in clinical practice and has strong prognostic value in patients with AD. Notably, low BP values at presentation are associated with higher mortality (10, 11), even though a J-curve relationship between systolic BP (SBP) and in-hospital mortality has been also suggested in alternative AD populations (12). Conversely, it is unknown whether the impact of BP at presentation on prognosis is influenced by a previous diagnosis of hypertension. In this regard, the use of antihypertensive medications is reported to influence the prognosis in patients with AD (4, 13, 14) and therefore could constitute a potential confounding factor when assessing the relationship between BP at presentation and mortality. In addition, whether BP at presentation could also be a marker of alternative organ damage in patients with AD remains to be established.

This study evaluated a sample of patients with AD who performed echocardiograms at the time of AD diagnosis and compared clinical, cardiac, and prognostic characteristics between those with (HypHist) and without (No-HypHist) hypertension history and further evaluated the association of BP at presentation with LV remodeling, renal dysfunction, and 1-year mortality.

## Methods

### Study Population

This study retrospectively evaluated individuals with AD who performed echocardiogram exams within 60 days after or before the diagnosis of AD and were managed at 3 Brazilian centers [Clinics Hospital of the University of Campinas, Cardiology Emergency Room of Pernambuco (PROCAPE), and Hospital of the Pontifical Catholic University of Campinas] from 1993 to 2020. Exclusion criteria were as follows: (1) AD of traumatic origin; (2) prior AD; (3) age <18 years; (4) moderate or severe valvar disease except for aortic valve regurgitation; and (5) LV remodeling due to previous myocardial infarction. Originally, 696 individuals with AD diagnosis were identified, but 329 were excluded due to having no available echocardiogram (*n* = 251), performing an echocardiogram exam more than 60 days before or after AD (*n* = 71), or fulfilling the exclusion criteria (*n* = 7), leaving 367 for the current analysis. The study protocol was approved by the Ethics Committee of all participant centers, which waived the requirement for informed consent.

### Clinical Variables

The diagnosis of AD was performed by multislice CT angiography imaging or transesophageal echocardiography. AD involving the ascending aorta and/or aortic arch was defined as Type-A, while AD with an entry tear beyond the left subclavian artery origin, and sparing the ascending aorta and aortic arch, was defined as Type-B. Information on clinical presentation and medical history at the time of AD diagnosis was thoroughly obtained from medical charts and comprised the following data: sex, age, body mass index, SBP, and diastolic BP (DBP) obtained at presentation at the arm with the highest value, presence of hypotension (SBP < 90 mmHg) (2), any pain (chest, back, or abdominal) at presentation, any limb deficit, pleural effusion, cardiac tamponade, creatinine levels, AD presentation [acute (symptom onset up to 7 days), subacute (symptom onset 8–30 days) and chronic (symptom onset >30 days or asymptomatic)] (2), AD extension, and history of hypertension, diabetes mellitus, ever smoking, Marfan syndrome, coronary heart disease, and use of antihypertensive medications. Pulse pressure was calculated as follows: SBP—DBP. Individuals who self-reported a diagnosis of hypertension or use of antihypertensive medications were defined as having hypertension history and were labeled as HypHist, while those without hypertension history were labeled as No-HypHist. Information reported by HypHist participants regarding hypertension control prior to the AD event was also gathered from the charts. Diabetes was defined based on self-reported diagnosis or reported use of antidiabetic medications, and coronary heart disease was defined as a history of previous myocardial infarction or documentation of cardiac ischemia by non-invasive tests (stress echocardiography, myocardial perfusion scintigraphy, or exercise test) or coronary angiography. In-hospital data were collected and included information on the modality of definitive treatment used to manage AD (surgery, endovascular therapy, or medical therapy), descending aorta stent placement (solely for patients with Type-A who underwent surgery), and aortic valve replacement. The estimated glomerular filtration rate (eGFR) was calculated by the Cockroft-Gault formula and then normalized to a body surface area of 1.73 m^2^. Decreased eGFR was considered if <60 ml/min/1.73 m^2^.

### Echocardiography

All enrolled patients performed a transthoracic echocardiography exam within 60 days after or before AD diagnosis. This time range was chosen because substantial changes in LV structure due to therapeutic interventions were assumed to not occur within that period. The median (25th, 75th percentiles) time between the date when echocardiogram was performed and the date of AD diagnosis was 1 (0, 6) days. Two-dimensional echocardiography was performed as previously described (15–17), in accordance with the American Society of Echocardiography recommendations (18). LV mass was indexed by body surface area, and relative wall thickness was calculated as 2 × posterior wall thickness/LV diastolic diameter. LV hypertrophy was defined as LV mass index >95 and 115 g/m^2^ in women and men, respectively, while LV concentricity was defined as wall thickness >0.42 (18). The following LV geometric patterns were defined: normal geometry (no LV hypertrophy or LV concentricity), eccentric hypertrophy (LV hypertrophy without LV concentricity), concentric hypertrophy (LV hypertrophy with LV concentricity), and concentric remodeling (LV concentricity without LV hypertrophy) (5). LV ejection fraction (LVEF) was calculated by the Teicholz method.

### Outcomes

The primary outcome was all-cause death up to 1-year post-AD diagnosis. The follow-up was assessed by last hospital visit or telephone contact. Death was ascertained by medical record review or by the national social security number database. All patients whose death was ascertained by medical record analysis had the cause of death established (*n* = 100). Among patients whose death was certificated by the national social security number database (*n* = 12), the cause of death was not available and therefore was defined as unknown.

### Statistical Analysis

Continuous variables with normal and non-normal distribution and categorical variables are presented as mean ± SD, median (25th, 75th percentiles), and numbers (proportions), respectively. Differences in studied variables were evaluated by unpaired *t*-test or one-way ANOVA for normally distributed variables, Mann-Whitney or Kruskal-Wallis test for non-normally distributed variables, and χ^2^-test for categorical variables, according to the type of the variable and the number of studied groups. Multivariable logistic regression and restricted cubic spline analyses adjusted for age, sex, center, body mass index, and variables that might potentially influence the cardiac structure and kidney function [HypHist, previous use of antihypertensive medications, diabetes mellitus, AD type, and aortic regurgitation grade (solely for echocardiography data)] evaluated the association of BP components (SBP, DBP, and pulse pressure) with LV hypertrophy, LV concentricity, and eGFR <60 ml/min/1.73 m^2^, and the likelihood ratio test was used to assess interactions by HypHist status or AD type. Kaplan-Meier method was used to calculate cumulative event rate, and comparisons between the curves were made by log-rank test. Multivariable Cox regression models were used to evaluate the association of HypHist with mortality adjusted for age, sex, center, calendar time, history of controlled hypertension prior to AD, and variables that were statistically different between patients who were dead or not at 1-year of follow-up. Further multivariable Cox regression analyses were performed to evaluate the association between LVEF and mortality. The relationship between BP at presentation and 1-year mortality in the whole sample and according to HypHist status was assessed by adjusting restricted cubic splines with 4 knots, and the likelihood ratio test was used to assess interactions by HypHist status. As a sensitivity analysis, we evaluated the characteristics and 1-year mortality of the participants by dividing the total sample into 3 groups: (1) No-HypHist patients with normal BP (<140/90 mmHg) at presentation; (2) No-HypHist patients with elevated BP (≥140/90 mmHg) at presentation; and (3) patients with HypHist. In addition, we compared the association between BP at presentation and 1-year mortality by adjusting restricted cubic splines according to previous use of antihypertensive medications in the HypHist group and according to HypHist status and AD type in the whole sample. Statistical analysis was performed using Stata software V.14.2 (Stata Corp LP, College Station, TX, USA). *P*-values of <0.05 were considered statistically significant.

## Results

### Clinical and Echocardiographic Characteristics According to Hypertension Status

The total sample comprised 367 individuals (70% men, 57.0 ± 12.3 years, 66% with Type-A AD), among whom 81% had a history of hypertension (HypHist). Data on clinical presentation, medical history, and in-hospital procedures split by HypHist status are shown in Table 1. Patients with HypHist tended to be older and were more likely to have greater SBP, DBP, and body mass index, to undergo medical therapy, while patients with No-HypHist were more likely to present hypotension and acute AD, to have Marfan syndrome, and to undergo surgical therapy and aortic valve replacement. Echocardiography and kidney function data split by HypHist status are shown in Table 2. Patients with HypHist had greater LV mass index and relative wall thickness and were more likely to have LV hypertrophy, LV concentricity, concentric hypertrophy, and worse kidney function, while patients with No-HypHist were more likely to have normal LV geometry and bicuspid aortic valve.

**Table 1:** Clinical and in-hospital characteristics according to hypertension status.

**Variables**	**No-HypHist**	**HypHist**	***p*-value**
*N* (%)	69 (19)	298 (81)	
**Clinical presentation**			
Male sex, *n* (%)	54 (78)	202 (68)	0.09
Age, years	54.7 ± 13.8	57.5 ± 11.9	0.08
Type-A AD, *n* (%)	49 (71)	193 (65)	0.32
Systolic BP, mmHg	132.8 ± 35.8	151.4 ± 39.0	<0.001
Diastolic BP, mmHg	75.0 ± 20.6	87.9 ± 24.5	<0.001
Pulse pressure, mmHg	57.8 ± 26.2	63.6 ± 25.4	0.09
Body mass index, kg/m^2^	24.9 ± 4.6	27.7 ± 5.2	<0.001
Any paint^*^, *n* (%)	55 (80)	241 (81)	0.83
Any limb pulse deficit, *n* (%)	23 (33)	95 (32)	0.82
Cardiac tamponade, *n* (%)	4 (6)	8 (3)	0.19
Hypotension, *n* (%)	5 (7)	7 (2)	0.037
Pleural effusion, *n* (%)	14 (20)	45 (15)	0.29
AD presentation, *n* (%)			0.021
Acute	61 (88)	216 (72)	
Subacute	3 (4)	29 (10)	
Chronic	5 (7)	53 (18)	
AD extension, *n* (%)			
Descending aorta (Type-A)	30 (61)	134 (69)	0.27
Abdominal aorta (Type-B)	18 (90)	85 (81)	0.33
**Medical history**			
Ever smoking, *n* (%)	23 (34)	117 (39)	0.39
Diabetes mellitus, *n* (%)	3 (4)	31 (10)	0.12
Coronary heart disease, *n* (%)	3 (4)	32 (11)	0.10
Marfan syndrome, *n* (%)	6 (9)	2 (1)	<0.001
Previous controlled hypertension, *n* (%)	——	104 (35)	——
Antihypertensive medication use, *n* (%)	0 (0)	230 (77)	<0.001
ACEI or ARB, *n* (%)	0 (0)	175 (59)	<0.001
Diuretic, *n* (%)	0 (0)	84 (28)	<0.001
Calcium channel blocker, *n* (%)	0 (0)	72 (24)	<0.001
Beta-blocker, *n* (%)	0 (0)	112 (38)	<0.001
**In-hospital data**			
Definitive treatment, *n* (%)			0.037
Medical therapy	7 (10)	67 (23)	
Endovascular	8 (12)	43 (14)	
Surgery	54 (72)	187 (63)	
AoV replacement (Type-A), *n* (%)	21 (30)	38 (13)	<0.001
Descending aorta stent (Type-A), *n* (%)	18 (37)	49 (25)	0.11

* *Chest, back, or abdominal pain*.

*AD, aortic dissection; AoV, aortic valve; BP, blood pressure; ACEI or ARB, angiotensin-converting enzyme inhibitor or angiotensin receptor blocker; HypHist, patients with history of hypertension; No-HypHist, patients without history of hypertension*.

**Table 2:** Echocardiography characteristics and kidney function of according to hypertension status.

**Variables**	**No-HypHist**	**HypHist**	***p*-value**
*N* (%)	69 (19)	298 (81)	
**Echocardiography**			
LV diastolic diameter, mm	53.0 ± 9.8	52.6 ± 8.0	0.72
Septum wall thickness, mm	10.8 ± 2.1	12.0 ± 2.6	<0.001
Posterior wall thickness, mm	10.6 ± 2.1	11.7 ± 2.3	<0.001
LV mass index, g/m^2^	129.9 ± 53.4	146.7 ± 59.7	0.033
Relative wall thickness	0.42 ± 0.13	0.45 ± 0.11	0.013
LV ejection fraction, %	63.2 ± 10.4	63.9 ± 10.6	0.66
LV ejection fraction <50%, *n* (%)	6 (9)	30 (10)	0.79
LV hypertrophy, *n* (%)	40 (58)	213 (72)	0.029
LV concentricity, *n* (%)	30 (44)	177 (59)	0.016
Normal geometry, *n* (%)	18 (26)	45 (15)	0.029
Concentric remodeling, *n* (%)	11 (16)	40 (13)	0.59
Concentric hypertrophy, *n* (%)	19 (28)	137 (46)	0.005
Eccentric hypertrophy, *n* (%)	21 (30)	76 (26)	0.40
Bicuspid aortic valve, *n* (%)	3 (4)	2 (1)	0.018
Aortic regurgitation grade, *n* (%)			0.09
No	28 (41)	158 (53)	
Mild	21 (30)	85 (29)	
Moderate/severe	20 (29)	55 (18)	
**Kidney function**			
eGFR, mL/min/1.73 m^2^	80.6 ± 37.1	67.7 ± 29.5	0.002
eGFR <60 mL/min/1.73 m^2^, n (%)	19 (28)	120 (41)	0.043

*LV, left ventricular; eGFR, estimated glomerular filtration rate; HypHist, patients with a history of hypertension; No-HypHist, patients without a history of hypertension*.

In the whole sample, 81% had pain (chest, back, or abdominal) at presentation, but there were no differences in BP values between those with and without pain (SBP = 149.1 ± 39.0 vs. 143.3 ± 32.8 mmHg, *p* = 0.26; DBP = 86.3 ± 24.7 vs. 82.2 ± 22.6 mmHg, *p* = 0.21).

### Relationship Between BP at Presentation and Cardiac and Renal Alterations

**Figure 1:**
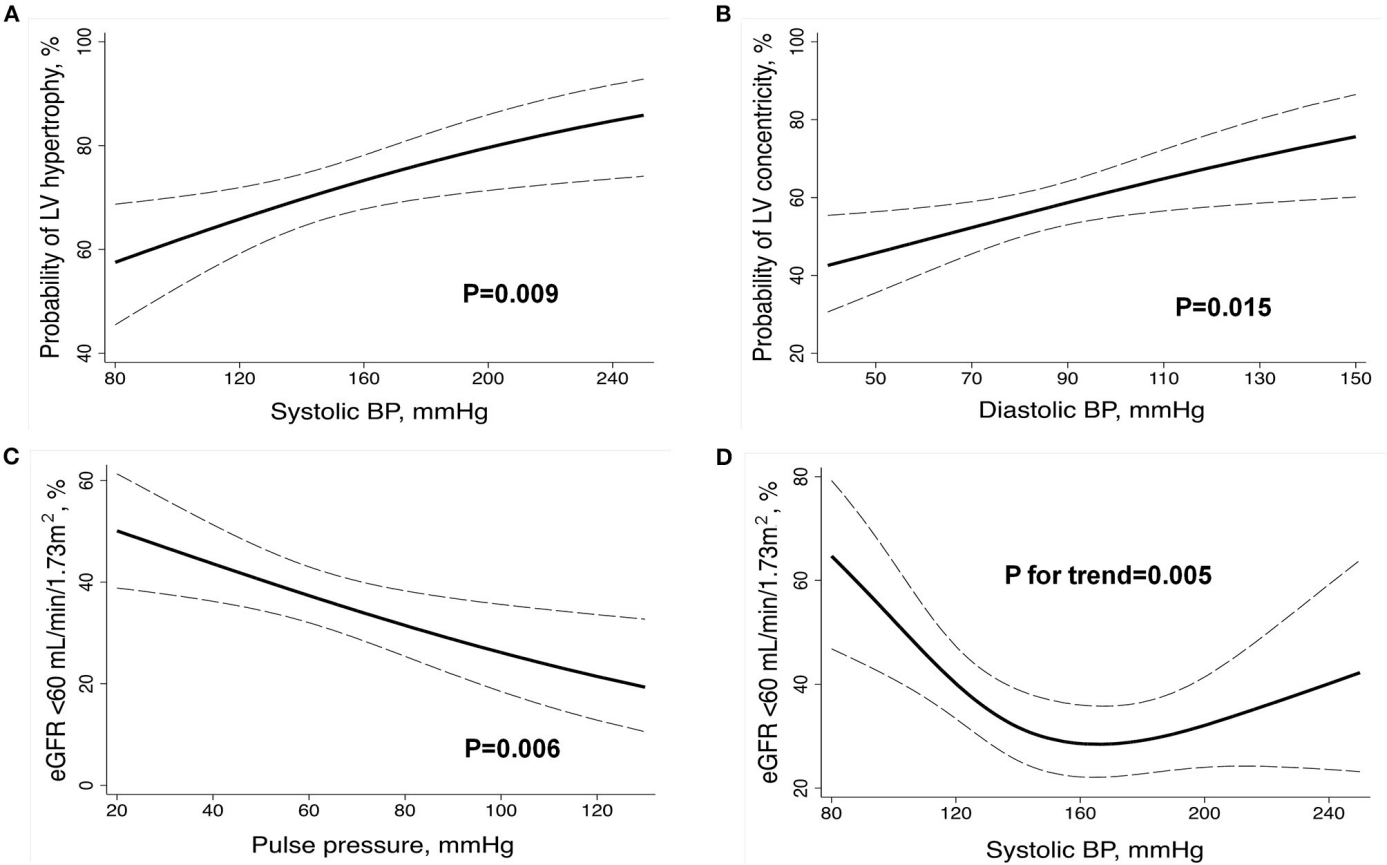
Relationship between BP components at presentation and markers of left ventricular remodeling and renal dysfunction in the whole sample. Multivariable logistic regression **(A–C)** and restricted cubic spline **(D)** analyses were adjusted for age, sex, center, body mass index, history of hypertension, previous use of antihypertensive medications, diabetes mellitus, aortic dissection type, and aortic regurgitation grade (solely for models evaluating LV variables). BP, blood pressure; eGFR, estimated glomerular filtration rate; LV, left ventricular. The dashed lines indicate the 95% CIs.

Logistic regression analysis adjusted for age, sex, center, body mass index, HypHist status, previous use of antihypertensive medications, diabetes mellitus, AD type, and aortic regurgitation grade (solely for models evaluating LV variables) evaluated the association of BP components (SBP, DBP, and pulse pressure) at presentation with LV hypertrophy, LV concentricity, and eGFR <60 ml/min/1.73 m^2^ (Supplementary Table S1). LV hypertrophy had a direct and more significant association with SBP, while LV concentricity was directly associated with DBP, and eGFR <60 ml/min/1.73 m^2^ was inversely associated with pulse pressure (Figures 1A–C; Supplementary Table S1). We further evaluated whether there was a non-linear relationship between BP components and cardiac or renal alterations and found a J-curve association between SBP and eGFR <60 ml/min/1.73 m^2^, with a prominent and inverse relationship at low SBP levels (Figure 1D). No significant interactions for the aforementioned associations were detected according to AD type or HypHist status, except for a greater association between pulse pressure and reduced eGFR in patients with No-HypHist (Supplementary Figure S1).

### Outcomes

After 1 year of follow-up, there were 90 (30%) and 22 (32%) deaths among patients with and without hypertension, respectively. There was no impact of hypertension status on 1-year mortality as assessed by Kaplan-Meier analysis (Supplementary Figure S2) and on the causes of death (Supplementary Table S2). Patients who were dead at 1 year of follow-up were more likely to have Type-A AD, hypotension, acute AD, and lower SBP and pulse pressure at presentation and were less likely to be previously using use beta-blockers and to undergo endovascular therapy (Supplementary Table S3). In addition, patients who died during follow-up were more likely to have LV concentricity, concentric remodeling, and worse kidney function and were less likely to have normal LV geometry at baseline (Supplementary Table S4).

**Figure 2:**
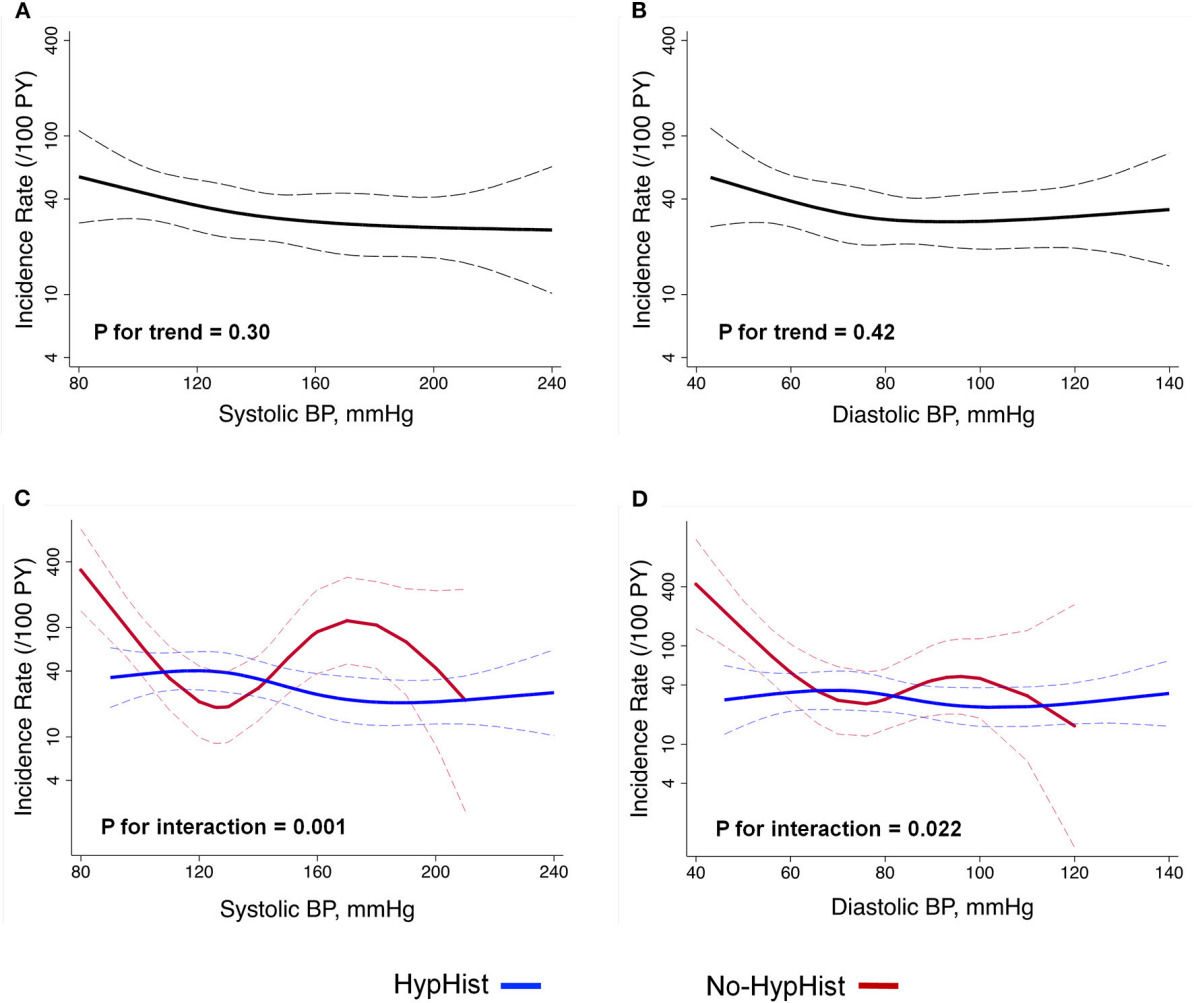
Relationship between BP at presentation and 1-year mortality. Analyses were adjusted for age, sex, center, calendar time, history of controlled hypertension prior to AD, aortic dissection type, in-hospital treatment modality, aortic dissection presentation, estimated glomerular filtration rate, previous beta-blocker use, left ventricular geometric patterns, and HypHist status. Analysis includes the whole sample **(A,B)** and is split by HypHist status **(C,D)**. **(C,D)** HypHist status was not included as a covariate. BP, blood pressure; HypHist, patients with a history of hypertension; No-HypHist, patients without a history of hypertension. The dashed lines indicate the 95% CIs.

Results of multivariable Cox-regression analysis adjusted for age, sex, center, calendar time, history of controlled hypertension prior to AD, AD type, in-hospital treatment modality, AD presentation, eGFR, previous beta-blocker use, and LV geometric patterns confirmed no association between hypertension and 1-year mortality [hazard ratio (HR), 0.66; 95% CI, 0.39–1.14; *p* = 0.14]. We then evaluated the relationship between BP components and 1-year mortality by multivariable restricted cubic splines analysis adjusted for age, sex, center, calendar time, history of controlled hypertension prior to AD, AD type, in-hospital treatment modality, AD presentation, eGFR, previous beta-blocker use, LV geometric patterns, and HypHist status (Figure 2). There was no significant association between 1-year mortality and SBP and DBP in the whole sample (Figures 2A,B). Conversely, there were significant interactions for the relationship between BP and mortality according to HypHist status (Figures 2C,D). While SBP and DBP were not associated with death among patients with HypHist, these measures showed a J-curve association with 1-year mortality among patients with No-HypHist. In this latter group, there was a steep inverse association between BP and mortality, especially when SBP/DBP values were below ≈120/70 mmHg, while SBP was associated with higher mortality when >140 mmHg (Figures 2C,D). We then compared the causes of death between HypHist and No-HypHist according to normal BP (SBP < 140 mmHg and DBP < 90 mmHg) and elevated BP (SBP ≥ 140 mmHg and/or DBP ≥ 90 mmHg) at presentation and found that the majority of deaths in patients with normal BP of both groups was due to cardiogenic/hypovolemic shock (Supplementary Table S5). No significant association between 1-year mortality and pulse pressure was detected in the whole sample or in patients with or without hypertension. In addition, there was no association between mortality and LVEF (HR, 0.99; 95% CI, 0.97–1.01; *p* = 0.46) or LVEF <50% (HR, 1.45; 95% CI, 0.79–2.67; *p* = 0.23) in multivariable Cox-regression analysis.

### Sensitivity Analysis

Given the direct association between BP and cardiac remodeling in the whole sample and the differences in the relationship between BP and mortality in No-HypHist patients with SBP lower and greater than 140 mmHg, we evaluated the characteristics of the participants by dividing the total sample into 3 groups: (1) No-HypHist patients with normal BP (<140/90 mmHg) at presentation (*n* = 33); (2) No-HypHist patients with elevated BP (≥140/90 mmHg) at presentation (*n* = 36); and (3) Patients with HypHist (*n* = 298; Supplementary Tables S6, S7). Group 1 was more likely to have cardiac tamponade, hypotension, Marfan syndrome, bicuspid aortic valve, and lower prevalence of LV hypertrophy and LV concentricity, while group 2 tended to have clinical characteristics and prevalence of LV hypertrophy and LV concentricity similar to group 3. Conversely, eGFR was lower in groups 1 and 3 as compared with group 2. In fully adjusted linear Cox-regression analysis, group 1 had similar 1-year mortality compared with group 2 (HR, 0.88; 95% CI, 0.37–2.07; *p* = 0.77) and group 3 (HR, 0.62; 95% CI, 0.31–1.25; *p* = 0.18), and group 2 had similar mortality compared with group 3 (HR, 1.42; 95% CI, 0.72–2.80; *p* = 0.32).

There were distinct associations between SBP at presentation and 1-year mortality in the HypHist group according to previous use of antihypertensive medications, with greater mortality in the SBP range of 140–200 mmHg among those not using antihypertensive medications (Supplementary Figure S3). Analyses evaluating the association between BP at presentation and mortality split by AD type showed results similar to the primary analysis, except for no interaction for the relationship between SBP and mortality according to HypHist status among patients with Type-B (Supplementary Figure S4).

## Discussion

This multicenter study evaluating patients with AD has 4 major findings. First, BP values at presentation showed a J-curve association with mortality among patients with No-HypHist but did not associate with death among patients with HypHist. Notably, most of the deaths among patients with low BP values were due to cardiogenic/hypovolemic shock. Second, the association between BP at presentation and mortality was influenced by previous use of antihypertensive medications in the HypHist group. Third, BP values at presentation were markers of adverse cardiac remodeling and altered renal function in the whole sample. Fourth, patients with HypHist had several distinct clinical and echocardiographic characteristics but a similar prognosis compared with patients with No-HypHist. These findings indicate that combined information on BP at presentation, previous diagnosis of hypertension, and use of antihypertensive medications might be useful to predict mortality risk and to estimate cardiac and renal alterations among patients with AD.

One major finding of this report was that BP at presentation was associated with 1-year mortality in No-HypHist, but not in patients with HypHist. In patients with No-HypHist, BP at presentation showed a J-curve relationship with mortality, with SBP/DBP <120/70 mmHg and SBP >140 mmHg showing greater association with mortality. In general, low BP values are an indication of AD complications associated with worse prognosis, such as cardiac tamponade, myocardial dysfunction, aortic rupture, or significant dysfunction of alternative organs that include the kidney, brain, and gut (12). In agreement with this assumption, No-HypHist patients with BP < 140/90 mmHg were more likely to present cardiac tamponade, renal impairment, and to undergo aortic valve replacement, and usually died of cardiogenic/hypovolemic shock. Conversely, the higher mortality risk associated with SBP > 140 mmHg might be related to a higher burden of hypertensive subclinical cardiovascular damage related to a worse prognosis among patients with AD, such as LV concentricity (4, 5), but could be also explained by AD progression due to greater aortic parietal stress eventually leading to rupture of the aorta and/or multiorgan failure. The hypothesis that No-HypHist patients with elevated BP at presentation were probably hypertensive individuals who were not aware of this condition was further supported by their clinical characteristics and prevalence of LV hypertrophy and LV concentricity, which were similar to those of patients with HypHist. In addition, our findings showing a J-curve association between BP at presentation and mortality are in agreement with data from the International Registry of Acute Aortic Dissection (IRAD) (12), which showed a J-curve association between SBP and in-hospital mortality in univariate analysis. However, our data are not able to provide insights into whether correcting hypotension or hypertension before surgical or endovascular treatment of AD can reduce long-term mortality risk.

The reasons for the lack of association between BP and mortality in the HypHist group were not clear in our analysis. However, because most patients with HypHist referred the use of antihypertensive medications, and antihypertensive medications were reported to influence mortality in patients with AD (4, 13, 14), it is possible that these pharmacological agents influenced the ability of BP at presentation to predict adverse prognosis. Indeed, we found that patients with HypHist using antihypertensive medications had lower 1-year mortality in the SBP range of 140–200 mmHg compared with those not using antihypertensive medications, strengthening the notion that previous use of antihypertensive medications may influence the impact of BP at presentation on prognosis.

In our analysis, SBP was associated with LV hypertrophy, while DBP was associated with LV concentricity, which reproduces results reported in an alternative hypertensive population (19). These data might have clinical implications, because LV hypertrophy and LV concentricity are acknowledged markers of long-term adverse cardiovascular events in general and hypertensive populations (5), and LV concentricity has been associated with greater mortality among patients with AD (4). Conversely, only 31% of No-HypHist patients with BP < 140/90 mmHg had normal LV geometry, indicating that mechanisms other than elevated BP, such as alterations in aortic stiffness and aortic valve regurgitation (4, 20, 21), might be involved in AD-associated LV remodeling, albeit it cannot be discarded that they previously had hypertension and were unaware of such diagnosis. We also found that lower pulse pressure was associated with worse renal function, which reproduces data obtained in patients with acute coronary syndrome and decompensated heart failure (22, 23). Furthermore, we found a J-curve association between SBP and eGFR < 60 ml/min/1.73 m^2^, with a marked inverse relationship at low SBP values. Together, these data suggest that low BP values at admission may be a marker of renal dysfunction among patients with AD, perhaps by reflecting inadequate kidney perfusion due to hemodynamic instability (24).

Consistent with available data (7, 8), we confirmed that a previous diagnosis of hypertension was not associated with differences in long-term mortality among patients with AD. Our analysis also provided some novel clues regarding potential explanations for the similar prognosis between patients with HypHist and No-HypHist. While patients with HypHist were less likely to receive surgical therapy and had a higher rate of LV concentricity and renal dysfunction, patients with No-HypHist were more likely to present hypotension and acute AD at presentation. These findings suggest that HypHist and No-HypHist groups had distinct clusters of characteristics related to a worse prognosis (2, 4), which might have eventually contributed to the balance of the mortality between the groups.

Some limitations of this report must be acknowledged. This is a retrospective and observational study based on chart review, which solely included patients who performed echocardiograms. Therefore, the present results may not be generalizable to all patients with AD, and the influence of residual confounding, selection bias, and missing data regarding previous hypertension diagnosis, and use of antihypertensive medications on our findings cannot be discarded. Patients with more severe diseases may have died before reaching the hospitals, thus altering the rate of patients with BP values at higher risk of adverse outcomes. Furthermore, data on the severity of hypertension in hypertensive patients prior to the event were not available.

## Conclusion

Blood pressure values at presentation had a J-curve association with mortality among patients with No-HypHist AD but did not associate with mortality among patients with HypHist AD. Notably, the lack of association between BP at presentation and mortality in patients with HypHist appears to be influenced by previous use of antihypertensive medications. Conversely, BP values at presentation were markers of adverse cardiac remodeling and altered renal function in the whole sample. These findings indicate that combined information on BP at presentation, previous diagnosis of hypertension, and use of antihypertensive medications might be useful to predict mortality risk and to estimate cardiac and renal alterations among patients with AD.

## Data Availability Statement

The raw data supporting the conclusions of this article will be made available by the authors, without undue reservation.

## Ethics Statement

The studies involving human participants were reviewed and approved by Ethics Committees of the State University of Campinas, Cardiology Emergency Room of Pernambuco and Pontifical Catholic University of Campinas. Written informed consent for participation was not required for this study in accordance with the national legislation and the institutional requirements.

## Author Contributions

MO, WR, and WN contributed to the design of the work, the acquisition, analysis and interpretation of data, and drafted the manuscript. JS, VL'A, MM, AR, AF, RL, PO, LS-F, OC-F, JM-S, OP, and AS contributed to the acquisition, analysis and/or interpretation of data, and revised the manuscript critically for important intellectual content. All authors gave final approval and agree to be accountable for all aspects of work ensuring integrity and accuracy.

## Funding

The study was supported by a grant from the Brazilian National Council for Scientific and Technological Development (CNPq; Grant 306154/2017-0), and São Paulo Research Foundation (FAPESP 2013/07607-8) for WN.

## Conflict of Interest

The authors declare that the research was conducted in the absence of any commercial or financial relationships that could be construed as a potential conflict of interest.

## Publisher's Note

All claims expressed in this article are solely those of the authors and do not necessarily represent those of their affiliated organizations, or those of the publisher, the editors and the reviewers. Any product that may be evaluated in this article, or claim that may be made by its manufacturer, is not guaranteed or endorsed by the publisher.

## Abbreviations

AD: aortic dissection
BP: blood pressure
eGFR: estimated glomerular filtration rate
HypHist: patients with a history of hypertension
LV: left ventricular
No-HypHist: patients without a history of hypertension.
