# Supplementary material for: Impact of Hypertension History and Blood Pressure at Presentation on Cardiac Remodeling and Mortality in Aortic Dissection
Source: Front Cardiovasc Med. 2022 Jan 21;8:803283. doi: 10.3389/fcvm.2021.803283 (PMC8813851; doi:10.3389/fcvm.2021.803283)
Supplement: Supplementary file 1 [file Data_Sheet_1.docx]

**SUPPLEMENTAL MATERIAL**

**Supplemental Table 1**. Adjusted logistic regression analysis between blood pressure at presentation and cardiac and renal alterations.

| BP component | Left ventricular hypertrophy | p-value | Left ventricular concentricity | p-value | eGFR  <60 mL/min/1.73m^2^ | p-value |
| --- | --- | --- | --- | --- | --- | --- |
| Systolic BP, mmHg | 1.009 [1.002-1.016] | 0.009 | 1.005 [0.999-1.011] | 0.09 | 0.994 [0.988-1.001] | 0.07 |
| Diastolic BP, mmHg | 1.011 [0.999-1.022] | 0.053 | 1.013 [1.002-1.024] | 0.015 | 1.000 [0.990-1.010] | 0.99 |
| Pulse pressure, mmHg | 1.011 [1.001-1.021] | 0.030 | 1.002 [0.993-1.010] | 0.73 | 0.987 [0.978-0.997] | 0.008 |

Data are presented as odds ratio and 95% confidence interval.

All analyses were adjusted for age, sex, center, body mass index, diabetes, aortic dissection type, hypertension history and previous use of antihypertensive medications. Aortic regurgitation grade was also included as independent variable in models assessing left ventricular hypertrophy or concentricity.

BP – blood pressure; eGFR – estimated glomerular filtration rate.

**Supplemental Figure 1.** Adjusted logistic regression analysis between pulse pressure at presentation and renal dysfunction according to HypHist status.


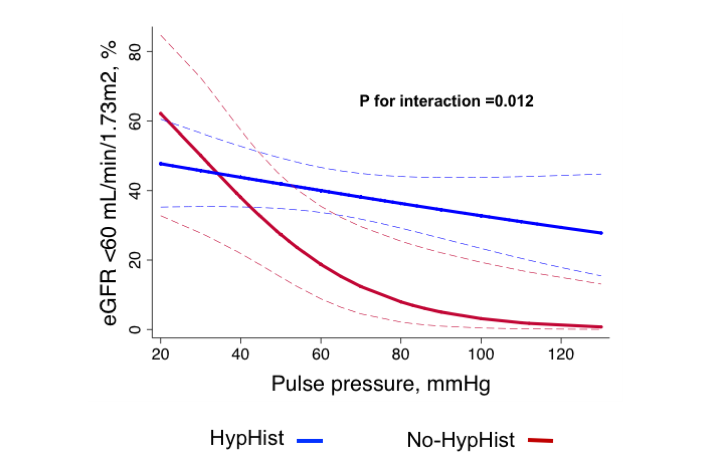


Analyses were adjusted for age, sex, center, body mass index, previous use of antihypertensive medications, diabetes mellitus, and AD type.

eGFR – estimated glomerular filtration rate; HypHist – patients with history of hypertension; No-HypHist – patients without history of hypertension.

The dashed lines indicate the 95% confidence intervals.

**Supplemental Figure 2.** Kaplan Meier curves for 1-year mortality in HypHist and No-HypHist patients.


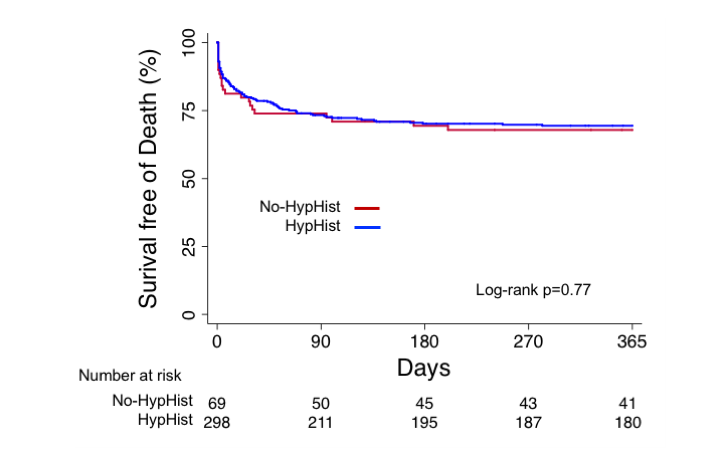


**Legend.** HypHist – patients with history of hypertension; No-HypHist – patients without history of hypertension.

**Supplemental Table 2.** Causes of death according to history of hypertension.

| Causes of Death | No-HypHist  (n=22) | HypHist  (n=90) | p-value |
| --- | --- | --- | --- |
| Cardiogenic/hypovolemic shock, n (%) | 10 (45) | 51 (57) | 0.34 |
| Cardiogenic shock, n (%) | 4 (18) | 20 (22) | 0.68 |
| Hypovolemic shock, n (%) | 6 (27) | 31 (34) | 0.52 |
| Stroke, n (%) | 2 (9) | 7 (8) | 0.84 |
| Sepsis, n (%) | 3 (14) | 14 (16) | 0.82 |
| Multiorgan failure, n (%) | 4 (18) | 8 (9) | 0.21 |
| Unknown, n (%) | 3 (14) | 9 (10) | 0.62 |

HypHist – patients with history of hypertension; No-HypHist – patients without history of hypertension.

**Supplemental Table 3.** Clinical characteristics of patients who were dead or not at 1 year of follow-up

| Variables | Survived | Deceased | p-value |
| --- | --- | --- | --- |
| *N (%)* | 255 (69) | 112 (31) |  |
| *Clinical Presentation* | |  |  |
| Male sex, n (%) | 181 (71) | 75 (67) | 0.44 |
| Age, years | 56.3 ± 12.0 | 58.5 ± 12.9 | 0.12 |
| Type-A AD, n (%) | 158 (62) | 84 (75) | 0.015 |
| Systolic BP, mmHg | 151.0 ± 38.5 | 141.0 ± 39.6 | 0.023 |
| Diastolic BP, mmHg | 86.8 ± 23.5 | 82.5 ± 26.0 | 0.12 |
| Pulse pressure, mmHg | 64.3 ± 25.9 | 58.5 ± 24.5 | 0.047 |
| Body mass index, kg/m^2^ | 27.3 ± 5.4 | 27.0 ± 4.6 | 0.60 |
| Any pain*, n (%) | 204 (80) | 92 (82) | 0.63 |
| Any limb pulse deficit, n (%) | 83 (33) | 35 (31) | 0.81 |
| Cardiac tamponade, n (%) | 6 (2) | 6 (5) | 0.14 |
| Hypotension, n (%) | 5 (2) | 7 (6) | 0.034 |
| Pleural effusion, n (%) | 41 (16) | 18 (16) | 1.00 |
| AD presentation, n (%) | |  | 0.022 |
| Acute | 182 (71) | 95 (85) |  |
| Subacute | 47 (18) | 11 (10) |  |
| Chronic | 26 (10) | 6 (5) |  |
| AD extension, n (%) | |  |  |
| Descending aorta (Type-A) | 101 (64) | 63 (75) | 0.08 |
| Abdominal aorta (Type-B) | 79 (81) | 24 (86) | 0.60 |
| *Medical history* | |  |  |
| Ever smoking, n (%) | 103 (40) | 37 (34) | 0.22 |
| Diabetes mellitus, n (%) | 24 (9) | 10 (9) | 0.88 |
| Coronary heart disease, n (%) | 24 (9) | 11 (10) | 0.90 |
| Marfan syndrome, n (%) | 6 (2) | 2 (2) | 0.73 |
| Hypertension, n (%) | 208 (82) | 90 (80) | 0.78 |
| Previous controlled hypertension**, n (%) | 77 (37) | 27 (30) | 0.24 |
| Antihypertensive medication use, n (%) | 166 (65) | 64 (57) | 0.15 |
| ACEI or ARB, n (%) | 127 (50) | 48 (43) | 0.22 |
| Diuretic, n (%) | 55 (22) | 29 (26) | 0.36 |
| Calcium channel blocker, n (%) | 52 (20) | 20 (18) | 0.57 |
| Beta-blocker, n (%) | 86 (34) | 26 (23) | 0.044 |
| *In-hospital data* | |  |  |
| Definitive treatment, n (%) | |  | 0.001 |
| Medical therapy | 45 (18) | 29 (26) |  |
| Endovascular | 46 (18) | 5 (5) |  |
| Surgery | 164 (64) | 77 (69) |  |
| AoV replacement (Type-A), n (%) | 41 (16) | 18 (16) | 1.00 |
| Descending aorta stent (Type-A), n (%) | 46 (29) | 21 (25) | 0.50 |

* Chest, back or abdominal pain

** Only among participants with history of hypertension

AD – aortic dissection; AoV – aortic valve; BP – blood pressure; ACEI or ARB – angiotensin-converting enzyme inhibitor or angiotensin receptor blocker.

**Supplemental Table 4.** Cardiac and renal characteristics of patients who were dead or not at 1 year of follow-up

| Variables | Survived | | Deceased | p-value |
| --- | --- | --- | --- | --- |
| *N (%)* | 255 (69) | | 112 (31) |  |
| *Echocardiography* |  | |  |  |
| LV diastolic diameter, mm | 52.9 ± 8.3 | | 52.2 ± 8.4 | 0.51 |
| Septum wall thickness, mm | 11.6 ± 2.5 | | 12.1 ± 2.7 | 0.13 |
| Posterior wall thickness, mm | 11.3 ± 2.2 | | 11.8 ± 2.4 | 0.05 |
| LV mass index, g/m^2^ | 140.7 ± 53.6 | | 149.8 ± 69.2 | 0.18 |
| Relative wall thickness | 0.44 ± 0.12 | | 0.46 ± 0.11 | 0.09 |
| LV ejection fraction, % | 64.0 ± 10.1 | | 63.2 ± 11.4 | 0.54 |
| LV ejection fraction <50%, n (%) | 23 (9) | | 13 (12) | 0.40 |
| LV hypertrophy, n (%) | 176 (69) | | 77 (69) | 0.96 |
| LV concentricity, n (%) | 131 (51) | | 76 (68) | 0.003 |
| Normal geometry, n (%) | 51 (20) | | 12 (11) | 0.030 |
| Concentric remodeling, n (%) | 28 (11) | | 23 (21) | 0.015 |
| Concentric hypertrophy, n (%) | 103 (40) | | 53 (47) | 0.22 |
| Eccentric hypertrophy, n (%) | 73 (29) | | 24 (21) | 0.15 |
| Bicuspid aortic valve, n (%) | 2 (1) | | 3 (3) | 0.15 |
| Aortic regurgitation grade, n (%) | | | | 0.14 |
| No | | 134 (53) | 52 (46) |  |
| Mild | | 76 (30) | 30 (27) |  |
| Moderate/severe | | 45 (18) | 30 (27) |  |
| *Kidney function* | |  |  |  |
| eGFR, mL/min/1.73m^2^ | | 73.7 ± 32.2 | 62.1 ± 28.2 | 0.001 |
| eGFR <60 mL/min/1.73m^2^, n (%) | | 88 (35) | 51 (46) | 0.045 |

LV – left ventricular; eGFR – estimated glomerular filtration rate

**Supplemental Table 5.** Causes of death according to history of hypertension and BP levels at presentation.

| Causes of Death | Systolic BP <140 mmHg and  diastolic BP <90 mmHg | | | Systolic BP ≥140 mmHg and/or  diastolic BP ≥90 mmHg | | |
| --- | --- | --- | --- | --- | --- | --- |
|  | No-HypHist  (n=11) | HypHist (n=43) | p-value | No-HypHist  (n=11) | HypHist (n=47) | p-value |
| Cardiogenic/hypovolemic shock, n (%) | 7 (64) | 27 (63) | 0.96 | 3 (27) | 24 (51) | 0.15 |
| Cardiogenic shock, n (%) | 4 (36) | 10 (23) | 0.38 | 0 (0) | 10 (21) | 0.09 |
| Hypovolemic shock, n (%) | 3 (27) | 17 (40) | 0.45 | 3 (27) | 14 (30) | 0.87 |
| Stroke, n (%) | 0 (0) | 2 (5) | 0.47 | 2 (18) | 5 (11) | 0.49 |
| Sepsis, n (%) | 3 (27) | 6 (14) | 0.29 | 0 (0) | 8 (17) | 0.14 |
| Multiorgan failure, n (%) | 1 (9) | 6 (14) | 0.67 | 3 (27) | 2 (4) | 0.014 |
| Unknown, n (%) | 0 (0) | 2 (5) | 0.47 | 3 (27) | 7 (15) | 0.33 |

HypHist – patients with history of hypertension; No-HypHist – patients without history of hypertension.

**Supplemental Table 6.** Clinical characteristics of patients according to hypertension history status and BP at presentation.

| Variables | No-HypHist  and BP <140/90 mmHg | No-HypHist and BP ≥140/90 mmHg | HypHist | p-value |
| --- | --- | --- | --- | --- |
| *N (%)* | n=36 | n=33 | n=298 |  |
| Clinical Presentation |  |  |  |  |
| Male sex, n (%) | 28 (78) | 26 (79) | 202 (68) | 0.23 |
| Age, years | 53.8 ± 16.4 | 55.6 ± 10.5 | 57.5 ± 11.9 | 0.18 |
| Type-A AD, n (%) | 29 (81) | 20 (61) | 193 (65) | 0.13 |
| Systolic BP, mmHg | 107.2 ± 18.5 | 159.9 ± 29.0 | 151.4 ± 39.0 | <0.001 |
| Diastolic BP, mmHg | 63.5 ± 13.7 | 87.2 ± 19.8 | 87.9 ± 24.5 | <0.001 |
| Pulse pressure, mmHg | 43.7 ± 12.4 | 72.7 ± 28.7 | 63.6 ± 25.4 | <0.001 |
| Body mass index, kg/m^2^ | 24.1 ± 5.3 | 25.7 ± 3.5 | 27.7 ± 5.2 | <0.001 |
| Any pain*, n (%) | 27 (75) | 28 (85) | 241 (81) | 0.57 |
| Any limb pulse deficit, n (%) | 12 (33) | 11 (33) | 95 (32) | 0.97 |
| Cardiac tamponade, n (%) | 4 (11) | 0 (0) | 8 (3) | 0.015 |
| Hypotension, n (%) | 5 (14) | 0 (0) | 7 (2) | <0.001 |
| Pleural effusion, n (%) | 10 (28) | 4 (12) | 45 (15) | 0.12 |
| AD presentation, n (%) |  |  |  | 0.05 |
| Acute | 30 (83) | 31 (94) | 216 (72) |  |
| Subacute | 3 (8) | 2 (6) | 53 (18) |  |
| Chronic | 3 (8) | 0 (0) | 29 (10) |  |
| AD extension, n (%) |  |  |  |  |
| Descending aorta (Type-A) | 16 (55) | 14 (70) | 134 (69) | 0.30 |
| Abdominal aorta (Type-B) | 6 (86) | 12 (92) | 85 (81) | 0.58 |
| Medical history |  |  |  |  |
| Ever smoking, n (%) | 10 (29) | 13 (39) | 117 (39) | 0.46 |
| Diabetes mellitus, n (%) | 1 (3) | 2 (6) | 31 (10) | 0.26 |
| Coronary heart disease, n (%) | 2 (6) | 1 (3) | 32 (11) | 0.25 |
| Marfan syndrome, n (%) | 6 (17) | 0 (0) | 2 (1) | <0.001 |
| Previous controlled hypertension, n (%) | ––– | ––– | 104 (35) | ––– |
| ACEI or ARB, n (%) | 0 (0) | 0 (0) | 175 (59) | <0.001 |
| Diuretic, n (%) | 0 (0) | 0 (0) | 84 (28) | <0.001 |
| Calcium channel blocker, n (%) | 0 (0) | 0 (0) | 72 (24) | <0.001 |
| Beta-blocker, n (%) | 0 (0) | 0 (0) | 112 (38) | <0.001 |
| In-hospital data |  |  |  |  |
| Definitive treatment, n (%) |  |  |  | 0.15 |
| Medical therapy | 3 (8) | 4 (12) | 67 (23) |  |
| Endovascular | 4 (11) | 4 (12) | 43 (14) |  |
| Surgery | 29 (81) | 25 (76) | 187 (63) |  |
| AoV replacement (Type-A), n (%) | 14 (39) | 7 (21) | 38 (13) | <0.001 |
| Descending aorta stent (Type-A), n (%) | 9 (31) | 9 (45) | 49 (25) | 0.16 |

* Chest, back or abdominal pain

AD – aortic dissection; AoV – aortic valve; BP – blood pressure; ACEI or ARB – angiotensin-converting enzyme inhibitor or angiotensin receptor blocker; HypHist – patients with history of hypertension; No-HypHist – patients without history of hypertension.

**Supplemental Table 7.** Cardiac and renal characteristics of patients according to hypertension history status and BP at presentation.

| Variables | No-HypHist  and BP <140/90 mmHg | No-HypHist and BP ≥140/90 mmHg | HypHist | p-value |
| --- | --- | --- | --- | --- |
| *N (%)* | n=36 | n=33 | n=298 |  |
| *Echocardiography* |  |  |  |  |
| LV diastolic diameter, mm | 54.4 ± 11.6 | 51.5 ± 7.2 | 52.6 ± 8.0 | 0.33 |
| Septum wall thickness, mm | 10.4 ± 2.0 | 11.2 ± 2.2 | 12.0 ± 2.6 | <0.001 |
| Posterior wall thickness, mm | 10.2 ± 1.9 | 11.0 ± 2.3 | 11.7 ± 2.3 | <0.001 |
| LV mass index, g/m^2^ | 128.3 ± 61.8 | 131.7 ± 43.2 | 146.7 ± 59.7 | 0.10 |
| Relative wall thickness | 0.39 ± 0.12 | 0.44 ± 0.14 | 0.45 ± 0.11 | 0.009 |
| LV ejection fraction, % | 61.7 ± 12.7 | 64.9 ± 7.0 | 63.9 ± 10.6 | 0.42 |
| LV ejection fraction <50%, n (%) | 4 (12) | 2 (6) | 30 (10) | 0.75 |
| LV hypertrophy, n (%) | 18 (50) | 22 (67) | 213 (71) | 0.030 |
| LV concentricity, n (%) | 13 (36) | 17 (52) | 177 (59) | 0.024 |
| Normal geometry, n (%) | 11 (31) | 7 (21) | 45 (15) | 0.050 |
| Concentric remodeling, n (%) | 7 (19) | 4 (12) | 40 (13) | 0.59 |
| Concentric hypertrophy, n (%) | 6 (17) | 13 (39) | 137 (46) | 0.003 |
| Eccentric hypertrophy, n (%) | 12 (33) | 9 (27) | 76 (26) | 0.60 |
| Bicuspid aortic valve, n (%) | 2 (6) | 1 (3) | 2 (1) | 0.040 |
| Aortic regurgitation grade, n (%) | | |  | 0.18 |
| No | 15 (42) | 13 (39) | 158 (53) |  |
| Mild | 9 (25) | 12 (36) | 85 (29) |  |
| Moderate/severe | 12 (33) | 8 (24) | 55 (18) |  |
| *Kidney function* | |  |  |  |
| eGFR, mL/min/1.73m^2^ | 65 [49, 107] | 79 [67, 94] | 68 [46, 85] | 0.009 |
| eGFR <60 mL/min/1.73m^2^, n (%) | 15 (42) | 4 (12) | 120 (41) | 0.005 |

LV – left ventricular; eGFR – estimated glomerular filtration rate; HypHist – patients with history of hypertension; No-HypHist – patients without history of hypertension.

**Supplemental Figure 3.** Relationship between BP at presentation and 1-year mortality in patients with history of hypertension according to previous use of antihypertensive medications.


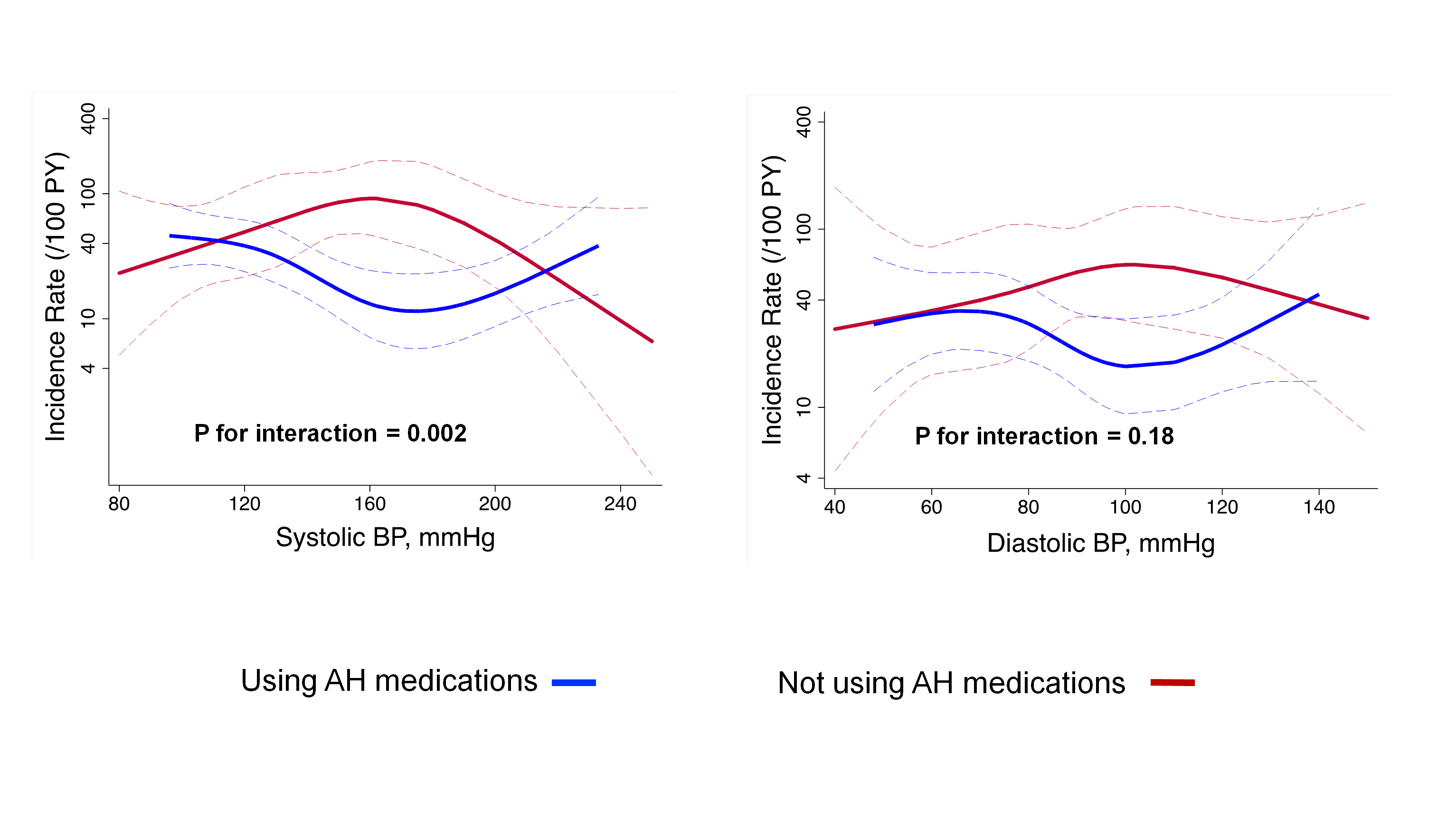


Analyses were adjusted for age, sex, center, calendar time, history of controlled hypertension prior to AD, aortic dissection type, in-hospital treatment modality, aortic dissection presentation, estimated glomerular filtration rate, previous beta-blocker use and left ventricular geometric patterns.

AH – antihypertensive; BP – blood pressure; HypHist – patients with history of hypertension; No-HypHist – patients without history of hypertension.

The dashed lines indicate the 95% confidence intervals.

**Supplemental Figure 4.** Relationship between BP at presentation and 1-year mortality according to aortic dissection type and history of hypertension status


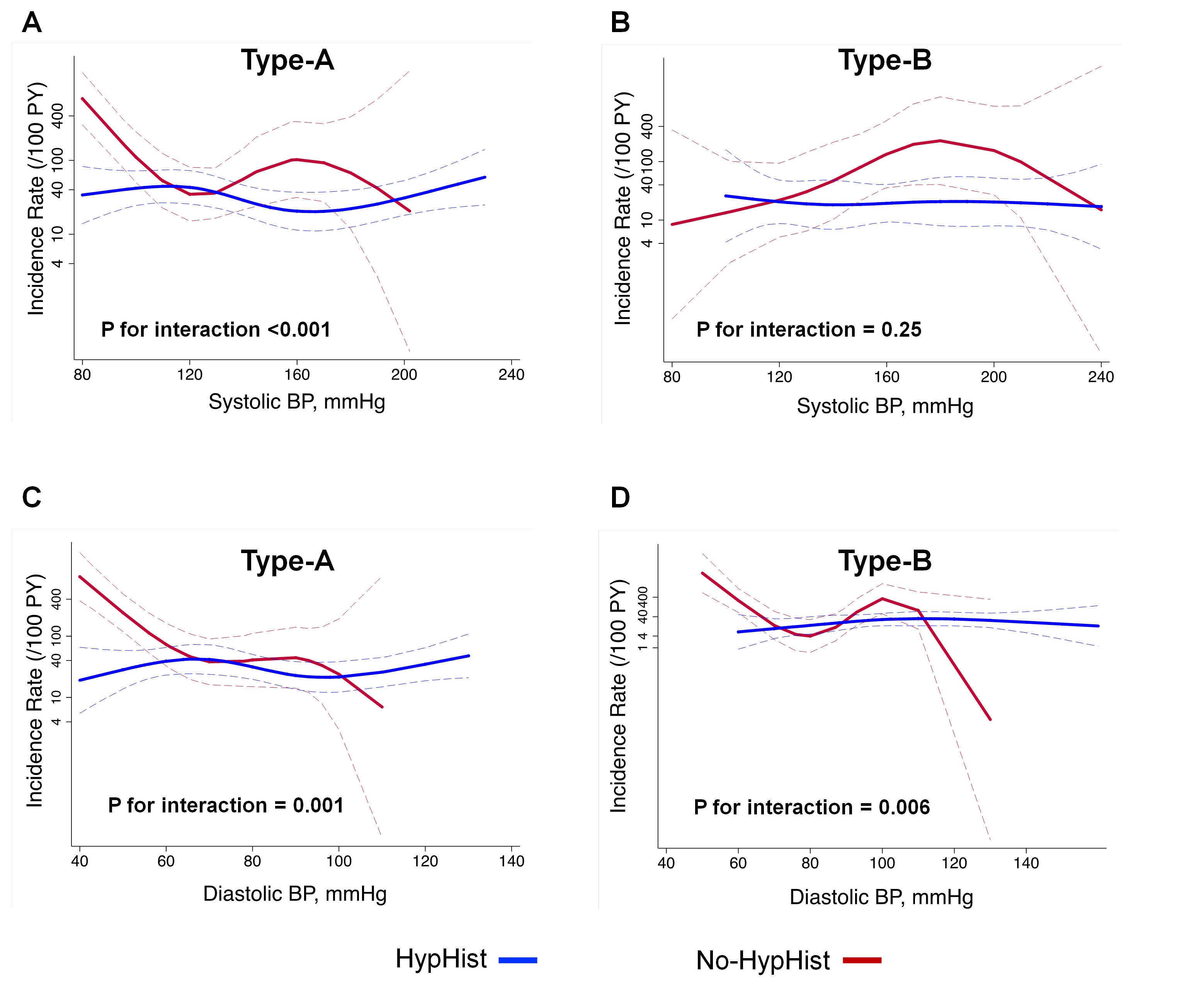


Analyses were adjusted for age, sex, center, calendar time, history of controlled hypertension prior to AD, in-hospital treatment modality, aortic dissection presentation, estimated glomerular filtration rate, previous beta-blocker use, and left ventricular geometric patterns.

BP – blood pressure; HypHist – patients with history of hypertension; No-HypHist – patients without history of hypertension.

The dashed lines indicate the 95% confidence intervals.
